# Model-Based Analysis of Context-Specific Cognitive Control

**DOI:** 10.3389/fpsyg.2012.00358

**Published:** 2012-09-24

**Authors:** Joseph A. King, Christopher Donkin, Franziska M. Korb, Tobias Egner

**Affiliations:** ^1^Center for Cognitive Neuroscience, Duke UniversityDurham, NC, USA; ^2^School of Psychology, University of New South WalesKensington, NSW, Australia

**Keywords:** cognitive control, conflict, evidence accumulation models, interference, mathematical modeling, priming, prediction error, response threshold

## Abstract

Interference resolution is improved for stimuli presented in contexts (e.g., locations) associated with frequent conflict. This phenomenon, the context-specific proportion congruent (CSPC) effect, has challenged the traditional juxtaposition of “automatic” and “controlled” processing because it suggests that contextual cues can prime top-down control settings in a bottom-up manner. We recently obtained support for this “priming of control” hypothesis with functional magnetic resonance imaging by showing that CSPC effects are mediated by contextually cued adjustments in processing selectivity. However, an equally plausible explanation is that CSPC effects reflect adjustments in response caution triggered by expectancy violations (i.e., prediction errors) when encountering rare events as compared to common ones (e.g., incongruent trials in a task context associated with infrequent conflict). Here, we applied a quantitative model of choice, the linear ballistic accumulator (LBA), to distil the reaction time and accuracy data from four independent samples that performed a modified flanker task into latent variables representing the psychological processes underlying task-related decision making. We contrasted models which differentially accounted for CSPC effects as arising either from contextually cued shifts in the *rate* of sensory evidence accumulation (“drift” models) or in the *amount* of evidence required to reach a decision (“threshold” models). For the majority of the participants, the LBA ascribed CSPC effects to increases in response threshold for contextually infrequent trial types (e.g., congruent trials in the frequent conflict context), suggesting that the phenomenon may reflect more a prediction error-triggered shift in decision criterion rather than enhanced sensory evidence accumulation under conditions of frequent conflict.

## Introduction

The ability to focus attention on information relevant to the task at hand while simultaneously ignoring myriad potential sources of distraction in the environment is critical for purposeful, goal-directed behavior. The efficiency at which the brain supports this ability to filter relevant stimuli from irrelevant noise can be gauged by “interference” effects in performance of classic selective attention/response conflict paradigms such as the Stroop color-word naming task (Stroop, 1935; MacLeod, 1991) or the Eriksen flanker task (Eriksen and Eriksen, 1974). In the flanker task, for instance, interference effects are expressed as reliably slower reaction times (RT) and decreased accuracy on trials in which a central target stimulus is flanked by incongruent distracters (e.g., HHSHH or (< > >) relative to trials in which the target is flanked by congruent ones (e.g., HHHHH or < < < < <). Interference (or “conflict”; defined as concurrent activation of mutually incompatible stimulus or response representations) is commonly thought to arise from involuntary, “automatic” processing of irrelevant information based on well-learned stimulus-response associations that are triggered in bottom-up fashion. Accordingly, the ability to resolve interference/conflict is thought to be dependent on effortful, “controlled” processing that employs internal goal representations to intentionally overcome habitual associations in a top-down manner (Cohen et al., 1990; Botvinick et al., 2001).

**Figure 1:**
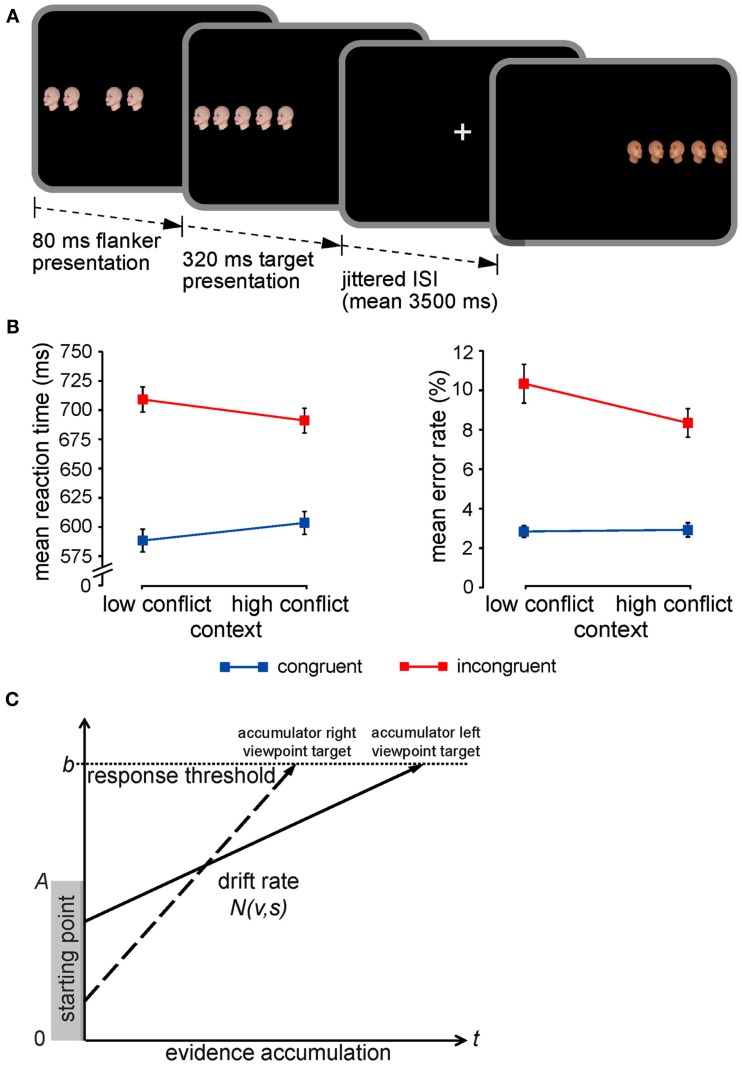
**Experimental paradigm, CSPC effects, and the LBA model**. **(A)** The face-viewpoint flanker task used to collect all four data sets was identical. Each trial began with the presentation of four novel (trial-unique) flanker faces, followed by an identical target face in the center of the array. Participants had to classify the viewpoint direction of the target face with a button press. Target and flanker face-viewpoint direction was congruent in half of all trials (shown here in the first trial) and incongruent in the other half (shown here in the second trial). The proportion of congruent to incongruent stimuli (conflict frequency) was manipulated in a context-specific manner according to stimulus location: one side of fixation was associated with 75% congruent trials (low-conflict context) and the other side with 75% incongruent trials (high-conflict context). For further details, see Section “Materials and Methods.” **(B)** Mean RTs and error rates (±SEM) are plotted for flanker congruent and incongruent trials as a function of the contextual conflict-frequency manipulation, illustrating the critical context × congruency interactions (i.e., CSPC effects). **(C)** The LBA model as applied to a typical decision in the face-viewpoint flanker task. One accumulator corresponds to the response that the target face is pointing left (solid arrow), while the other accumulator corresponds to a rightward response (dashed arrow). A response is triggered as soon as an accumulator reaches the response threshold, *b* (horizontal dotted line). Each accumulator begins with a starting amount of evidence drawn randomly from the range indicated by the gray-shaded rectangle (between 0 and *A*), and the accumulation rate (i.e., drift) for each response is drawn from a normal distribution with an appropriate mean, *v*, and SD, *s*.

Recent research using selective attention/response conflict tasks has challenged the traditional distinction between automatic and controlled processing, however, implying that this juxtaposition may in fact represent a false dichotomy. Specifically, several studies have suggested a melding of bottom-up associative processing and top-down attentional control settings by showing that when stimuli are presented in contexts (e.g., locations, colors, or sensory modalities) paired with frequent conflict, interference resolution is significantly improved (i.e., congruency effects are reduced; Corballis and Gratton, 2003; Crump et al., 2006, 2008; Lehle and Hübner, 2008; Wendt et al., 2008; Vietze and Wendt, 2009; Wendt and Kiesel, 2011; D’Angelo and Milliken, 2012; for review, see Bugg and Crump, 2012). Interestingly, these so-called context-specific proportion congruent (CSPC) effects occur even though observers are unaware of any systematic contextual variation in conflict frequency (Crump et al., 2008; Heinemann et al., 2009; Sarmiento et al., 2012). For example, using a modified Stroop task, Crump et al. (2006) showed that interference effects were reduced for stimuli presented in contexts (e.g., above central fixation) in which 75% of trials were incongruent (i.e., low proportion congruent/frequent conflict context) relative to those for stimuli presented in contexts (e.g., below fixation) in which 75% of trials were congruent (i.e., high proportion congruent/infrequent conflict context). The context-specificity and implicit nature of CSPC effects suggests that they are driven by bottom-up stimulus features. A purely associative explanation can be ruled out, however, because the context-specific improvement in interference resolution generalizes to frequency-unbiased stimuli (Crump and Milliken, 2009; Heinemann et al., 2009). Building on these previous findings, King et al. (2012) obtained neural evidence of bottom-up contextual priming of top-down control in a functional magnetic resonance imaging (fMRI) experiment. In particular, we found that the behavioral expression of CSPC effects in a flanker task variant using trial-unique stimuli (Figure 1A) was mirrored in contextual variation of hemodynamic activity associated with conflict processing in a region of the medial superior parietal lobule (mSPL) broadly implicated in top-down attentional selection (Yantis, 2008; Chiu and Yantis, 2009; Esterman et al., 2009; Greenberg et al., 2010; Shomstein, 2012) and that this activity explained modulation of stimulus-driven processing in task-relevant regions of sensory cortex.

Extant data pertaining to CSPC effects support the hypothesis that they reflect contextually cued adjustments in perceptual processing selectivity (e.g., Crump et al., 2006; Lehle and Hübner, 2008; Wendt et al., 2008; Crump and Milliken, 2009). That is, presentation of a stimulus in a context associated with frequent conflict appears to promote more efficient segregation of relevant from irrelevant stimulus information, facilitating faster responses to incongruent stimuli (but slower responses to congruent ones) relative to a context of infrequent conflict. However, an equally plausible alternative explanation is that the phenomenon is attributable to adjustments in response caution triggered by the relative frequency of events within each stimulus context. Specifically, a rare, contextually unlikely stimulus may induce a shift toward a more conservative response criterion, granting the observer more time for reaching a reliable perceptual decision. Thus, the characteristic pattern of CSPC effects (Figure 1B) could either reflect enhanced processing selectivity for stimuli presented in the frequent conflict context as suggested by several behavioral studies (e.g., Crump et al., 2006; Lehle and Hübner, 2008; Wendt et al., 2008; Crump and Milliken, 2009) and corroborated by our neuroimaging findings (King et al., 2012), or instead indicate a relative increase in response threshold when encountering unexpected, rare events (e.g., incongruent trials in the infrequent conflict context) as compared to expected or common ones (e.g., incongruent trials in the frequent conflict context). Neither conventional analyses of mean RT and error rates, nor our fMRI analyses could clearly disambiguate between these two possibilities. The purpose of the current study was to use a formal quantitative model of decision making to adjudicate between competing accounts of CSPC effects which differentially attribute the phenomenon to (1) contextually cued enhancement in processing selectivity or (2) shifts in response caution triggered by violations of expectancy regarding stimulus congruency (i.e., prediction error) within each context.

Quantitative sequential sampling models of decision making are increasingly being used to decompose the cognitive processes and neural mechanisms underlying choice RTs (for review, see Forstmann et al., 2011; Mars et al., 2012), such as those made in selective attention/response conflict paradigms (e.g., White et al., 2011). Several “evidence accumulation” models of choice have been developed (e.g., Smith and Vickers, 1988; Ratcliff and Rouder, 1998; van Zandt, 2000; Usher and McClelland, 2001; Brown and Heathcote, 2008), all of which vary in their assumptions regarding the precise nature of the constituent cognitive processes involved in rapid decision making and computational efficiency. Nonetheless, these models share the same basic notion that when participants make a decision about a stimulus, they continuously sample information from the environment and that this information serves as evidence for one of the possible responses. When evidence in favor of a potential response reaches a threshold, a decision is made and the associated response is given. In predicting performance, evidence accumulation models take into account the interaction between response speed and accuracy to estimate four central parameters: (1) an *a priori* bias for one or the other decision (“starting-point”), (2) the rate of evidence accumulation in favor of a particular decision (“drift rate”), (3) the amount of evidence that is necessary for triggering a particular decision (“response threshold”), and (4) the time involved in stimulus encoding and response execution (“non-decision time”). Here, we applied an established model of decision making, the linear ballistic accumulator (LBA) model (Brown and Heathcote, 2008; Donkin et al., 2009, 2011b; for examples of recent applications with comparable trial-per-condition numbers as in the current experiments, see e.g., Forstmann et al., 2008, 2010; Ho et al., 2009; Ludwig et al., 2009; van Maanen et al., 2011; McVay and Kane, 2012) to behavioral data collected from four independent samples during performance of the flanker task depicted in Figure 1A. Our objective was to test whether CSPC effects can be better accounted for as contextually cued shifts in the rate of evidence accumulation about the target stimulus (i.e., drift rate) or in the amount of evidence required to reach a decision (i.e., response threshold). Figure 1C illustrates how decisions regarding targets in this task are represented in the LBA. We predicted that if CSPC effects reflect contextually cued adjustments in processing selectivity, a model in which the rate of evidence accumulation (drift rate parameters) was allowed to vary across context and congruency conditions would provide the most parsimonious account of the empirical data (“drift” model). Alternatively, we expected that if CSPC effects reflect shifts in response caution triggered by unexpected, contextually unlikely stimuli, a model in which response threshold was allowed to vary across conditions would provide the best fit to the observed performance (“threshold” model).

## Materials and Methods

### Participants

The data reported here were collected from a total of 87 participants belonging to four independent samples that performed the identical flanker task (Figure 1A) in (1) the fMRI experiment described in King et al. (2012; *n* = 25; data set I), (2) a behavioral pilot study designed to test the adequacy of the paradigm for the magnetic resonance scanner environment (*n* = 19; data set II) and two follow-up behavioral studies designed to explore whether CSPC effects in this task, (3) are mediated by spatial stimulus-response compatibility effects (*n* = 25; data set III), or (4) vary as a function of awareness regarding the contextual conflict-frequency manipulation (*n* = 18; data set IV), respectively. All studies were conducted according to protocols approved by the Duke University Health System Institutional Review Board. For a detailed description of the sample contributing to data set I, see King et al. (2012). For data set II, a total of 21 volunteers with normal or corrected-to-normal vision participated. The data of two participants were excluded from further analysis due to chance level performance. The final sample (10 females, 9 males; mean age = 27.3 years; range = 22–37 years) included nine members of the Duke University Center for Cognitive Neuroscience (two research assistants, three doctoral students, three post-doctoral researchers, and one assistant professor) and 10 individuals recruited from the greater Durham, NC community by an advertisement on the Duke University Center for Cognitive Neuroscience Research Participation website who received $10 their participation. For data set III, a total of 26 undergraduates participated for class credit. The data of one participant was excluded from further analysis due to chance level performance. The final sample consisted of 17 females and 8 males (mean age = 20 years; range = 18–24 years). For data set IV, a total of 20 undergraduates participated for class credit. The data of two participants were excluded for chance level performance. The final sample consisted of 11 females and 7 males (mean age = 19.7 years; range = 18–23 years).

### Apparatus, stimuli, and procedure

Task programming, stimulus presentation, and behavioral recording were carried out with Presentation software (Neurobehavioral Systems; Albany, CA, USA). Face stimuli for the flanker experiment were generated with FaceGen software (Singular Inversions; Toronto, ON, Canada) to produce an equal number of left- and right-looking male and female faces (137 each; viewpoint angle: ∼45–50°) with unique identities from various age- and ethnic-groups. A total of 448 face images were used, one for each face trial of the experiment. Further details regarding stimulus generation are provided in King et al. (2012). For the fMRI experiment (data set I), stimuli were presented against a black background on a back projection screen, which participants viewed in a mirror mounted to the head coil; simulating a viewing distance of ∼80 cm. Given these viewing conditions, individual face stimuli within flanker arrays extended ∼0.72° horizontally and 1.1° vertically and were presented at ∼2.9, 3.8, 4.7, 5.6, and 6.5° horizontal visual angle to the left and right of central fixation. For the behavioral experiments (data sets II–IV), participants sat in a dimly lit room and viewed stimuli displayed against a black background on a 19″ LCD monitor at a distance of ∼80 cm, approximating the viewing conditions in the scanner.

In each trial of the flanker task (Figure 1A), a novel stimulus array (row of five identical trial-unique face images) was presented pseudorandomly either to the left or right of fixation. Participants were instructed to rapidly and accurately classify with a button press the viewpoint direction of the face in the center of the array (target) and ignore the flanker faces (distracters). For data sets I, II, and IV, responses were given with a right-hand index or middle finger button press. For data set III, responses were given with the index fingers of both hands. Stimulus-response mapping was counterbalanced across participants for all experiments. The target face was presented for 320 ms; its onset was delayed by 80 ms from the onset of the flanker faces, which were shown for 400 ms. Target and flanker face-viewpoint direction was congruent in half of all trials and incongruent in the other. Proportion congruency (i.e., conflict frequency) was manipulated according to stimulus location by defining one side of fixation as a high-conflict context (i.e., low proportion congruent; 25% congruent/75% incongruent trials) and the other as a low-conflict context (i.e., high proportion congruent; 75% congruent/25% incongruent trials; counterbalanced across participants). Inter-stimulus intervals were jittered between 3 and 5 s as randomly drawn from a pseudoexponential distribution, where 50% of intervals lasted 3 s, 25% lasted 3.5 s, 12% lasted 4 s, 6% lasted 4.5 s, and 6% lasted 5 s, resulting in a mean interval of ∼3.5 s. To counteract potential spatial stimulus-response compatibility effects in the fMRI experiment (data set I), participants responded on a response box (Current Designs, Philadelphia, PA, USA) that was vertically oriented on the participant’s chest (i.e., in plane with the length of their body). For the same reason, responses were given on the ↑ (8) and ↓ (2) buttons of the numeric keypad on a QWERTY US keyboard in the behavioral pilot experiment (data set II) and the experiment designed to test the influence of awareness of contextual variation in conflict frequency on CSPC effects (data set IV). Given that the purpose of data set III was to test whether CSPC effects might be mediated by potential stimulus-response compatibility effects, we asked participants to respond in a lateralized manner using the z and m keys on a QWERTY US keyboard. We explored the influence of awareness of the contextual conflict-frequency manipulation on CSPC effects in data set IV by informing the participants which side of fixation was associated with mostly congruent stimuli (low-conflict context) and mostly incongruent stimuli (high-conflict context) and encouraging them to use this information to their advantage. This manipulation was successful in that all 18 subjects that contributed to this data set reported that they noticed the location-based variation in congruency frequency in a post-test questionnaire, while only one out of 25 participants that contributed to data set I (King et al., 2012) reported explicit knowledge of the contextual conflict-frequency manipulation. Trials occurred in four blocks in the fMRI experiment (data set I; 112 trials each) and in seven blocks in the behavioral experiments (data sets II, III, and IV; 64 trials each). Participation in fMRI experiment lasted ∼75 min including a 64-trial training session, anatomical scanning, performance of an independent localizer task, and completion of a post-test survey (see King et al., 2012, for further details). Participation in the behavioral experiments lasted ∼40 min, including a 64-trial training session.

### Conventional analysis of response latency and accuracy

Prior to exploring the performance data with conventional analyses, we excluded the first trial of each block and all trials with excessively fast or slow responses (<150 ms/>2000 ms; 1.2% of all trials). We tested for contextual variation in interference effects [i.e., CSPC effects; (incongruent-congruent)_low-conflict context_ − (incongruent-congruent)_high-conflict context_] and their possible modulation as a function of spatial stimulus-response compatibility and/or awareness of the contextual conflict-frequency manipulation by submitting mean correct trial RTs (excluding post-error correct trials) and error rates to 2 (context: high conflict vs. low-conflict) × 2 (spatial stimulus-response compatibility: compatible vs. incompatible) × 2 (flanker congruency: congruent vs. incongruent) repeated-measures ANOVAs, using experimental session (data sets I–IV) as a between-subjects factor. Our previous fMRI study (data set I) revealed that CSPC effects varied as a function of context transitions. Specifically, they were only present for context repetitions, but absent for switches between contexts (e.g., from the low- to the high-conflict context; King et al., 2012). A supplementary 2 (context transition: repetition vs. switch) × 2 (context) × 2 (congruency) ANOVA using experimental session as a between-subjects factor explored whether this pattern was stable across data sets.

### Model fitting

The primary objective of the current study was to explore whether the LBA model attributes CSPC effects to contextually cued adjustments in processing selectivity (as indexed by the *rate* of evidence accumulation, i.e., drift) or to shifts in response caution triggered by unexpected stimuli within each context (as indexed by the *amount* of evidence required to make a decision, i.e., response threshold). These hypotheses were tested by fitting the performance data from each individual participant from each of the four data sets with models whose parameterizations reflected these differing assumptions about the influence of implicit contextual information on conflict processing. Support for each of the hypotheses comes from how well the respective models can fit the data. Readers unfamiliar with the methods involved in fitting sequential sampling models to choice RT data or the techniques involved in model selection (see the following section) are referred a tutorial paper which focuses specifically on the LBA, but is generally applicable to other evidence accumulation models (Donkin et al., 2011a).

We report the results of four models of CSPC effects in detail. The first two models (Models V1 and V2) assumed that CSPC effects arise from the influence of context on evidence accumulation rate (*v*; see Figure 1C). Both of these “drift” models accounted for CSPC effects by predicting the difference in *v* for congruent and incongruent stimuli to be larger in the low-conflict context than in the high-conflict context, but they did so in different ways. In Model V1, there was no constraint placed on *v*, and a separate parameter was estimated for each of the four experimental conditions (i.e., *v*_Incon-Low_, *v*_Con-Low_, *v*_Incon-High_, and *v*_Con-High_). In contrast, Model V2 assumed that the increase in *v* (i.e., faster rate) as we move from low- to high-conflict contexts for incongruent stimuli (recall that people get better at responding to incongruent stimuli in high-conflict contexts) is of the same magnitude as the decrease in *v* (i.e., slower rate) from low- to high-conflict contexts for congruent stimuli (people are worse for congruent stimuli in high-conflict contexts). As such, three rate parameters were estimated: *v*_Incon-Low_, *v*_Con-Low_, and Δ*v*, while accumulation rates in the high-conflict context were *v*_Incon-Low_ + Δ*v* for incongruent trials and *v*_Cong-Low_ − Δ*v* for congruent trials. In other words, Model V2 assumed the absolute difference in *v* resulting from a shift between contexts to be equal for congruent and incongruent trials. To illustrate, whereas a shift from the low- to the high-conflict context should lower *v* for congruent trials, it should increase *v* for incongruent trials to the same degree. In both drift models, response threshold was held constant across the high- and low-conflict contexts.

The latter two models (Models B1 and B2) assumed that CSPC effects arise from the influence of context on response threshold (*b*; see Figure 1C). Both of these “threshold” models accounted for CSPC effects by predicting that there would be differences in the distance from the top of the start-point distribution to response threshold, *b* − *A*. In particular, it was assumed that the difference between thresholds in the congruent and incongruent stimuli would be larger in the low-conflict than in the high-conflict context. However, as in the drift models outlined above, the threshold models also accounted for CSPC in different ways. In Model B1, as in Model V1, no constraint was placed on the way that response thresholds would change according to the context and congruency conditions, and so four threshold parameters were estimated (*b*_Incon-Low_ − *A*, *b*_Con-Low_ − *A*, *b*_Incon-High_ − *A*, and *b*_Con-High_ − *A*). In contrast, Model B2 was constrained in manner equivalent to Model V2 such that the absolute difference in response threshold for congruent and incongruent stimuli was equal between the low- and high-conflict contexts. That is, the reduction in thresholds as we move from low- to high-conflict contexts for incongruent stimuli is of the same magnitude as the increase in thresholds from low- to high-conflict contexts for congruent stimuli. In particular, three threshold parameters were estimated: *b*_Incon-Low_ − *A*, *b*_Con-Low_ − *A*, and Δ*b*, while thresholds in the high conflict were *b*_Incon-Low_ − *A* + Δ*b* for incongruent trials and *b*_Incon-Low_ − *A* − Δ*b* for congruent trials. In both threshold models, evidence accumulation rate was allowed to vary as a function of stimulus congruency, but not across the two contexts.

In all models, the SD of the distribution of drift rate across trials, *s*, the maximum of the uniform between-trial distribution of start-point, *A*, and non-decision time, *t*_0_, were fixed across the congruency and context conditions. Though no restrictions were made about the sign of Δ*b* and Δ*v* parameters, Models V2 and B2 were parameterized such that positive values of Δ*b* and Δ*v* would produce the standard CSPC effects.

Models were fit to each of the individual participants from each of the four data sets. The likelihood of the response time and response choice on each trial (the number of valid trials per participant after excluding response omissions ranged from 408 to 448; mean = 445 trials; SD = 5.8 trials) was calculated using the formulas outlined in Brown and Heathcote (2008). Particle swarm optimization was used to find best-fitting parameters by searching for the maximum of the sum of the likelihoods across all trials for each individual.

In addition to the models outlined above, we fit a number of other model parameterizations following standard practice (Donkin et al., 2011a) that we do not report here. For example, we fit one model in which both evidence accumulation rate and response thresholds were allowed to vary concurrently and another in which the CSPC effect was assumed to reflect a shift in non-decision time, *t*_0_. None of these models outperformed any of the models we report in detail, with probabilities generally not greater than about 5%. As such, we refrain from further discussion of these models.

### Model selection

The four models were compared using the commonly employed Akaike and Bayesian Information Criterion (AIC, Akaike, 1974; BIC, Schwarz, 1978, respectively). BIC was calculated using the standard formula

BIC=klnN-2lnL,

where *L* is the likelihood of the parameters given the data, *N* is the number of data points used to calculate the likelihood value, and *k* is the number of free parameters used to fit the data. Similarly, AIC was calculated using

AIC=2k-2lnL.

Note that for our data, AIC has a smaller complexity term whenever ln *N* > 8.

To aid interpretability, AIC and BIC values were converted into AIC and BIC weights using the method outlined in Wagenmakers and Farrell (2004). In short, the information criterion (IC) values are transformed in ΔIC values by subtracting the smallest IC value from the IC for each model. ΔIC are then turned into weights using the following

$$w_{i}\left(\text{IC}\right)=\frac{{\text{e}}^{-\frac{1}{2}\Delta_{i}\left(\text{IC}\right)}}{\underset{k}{\sum }{\text{e}}^{-\frac{1}{2}\Delta_{k}\left(\text{IC}\right)}}$$

where *w_i_* is the weight for the *i*th model. AIC and BIC weights reflect the probability that a particular model is true.

## Results

### Conventional analyses of response latency and accuracy

For the combined sample (*n* = 87), overall performance was high (93.5% correct) and characterized by typical flanker interference effects. RTs were slower for incongruent stimuli (700 ms) than for congruent arrays [596 ms; *F*(1,83) = 668.8; *p* < 0.0001]. Similarly, error rates were elevated on incongruent (9.2%) relative to congruent trials [3.0%; *F*(1,83) = 80.0; *p* < 0.0001]. Interestingly, a reversed spatial stimulus-response compatibility effect emerged in RTs. Responses were generally slower when the viewpoint direction of target faces (e.g., left) corresponded (i.e., were compatible) with the location of stimulus array (e.g., left of fixation; 655 ms) relative to when the viewpoint direction of targets did not correspond (i.e., were incompatible) with the stimulus position (641 ms). The magnitude of this effect varied across experimental sessions [*F*(3,83) = 2.9; *p* < 0.05] such that it was most pronounced in data set IV (22 ms), but virtually absent in data set I (3 ms). In any event, spatial stimulus-response compatibility effects did not interact with flanker congruency, stimulus context, or their combination [all *F*(3,83) < 3.3; n.s.] and therefore have no implications for the interpretation of the CSPC effects at the focus of interest in this study. The contextual manipulation of flanker conflict frequency did not have any general effect on RTs [*F*(1,83) = 0.3; n.s.], but error rates were elevated in the low- (6.5%) vs. high-conflict context [5.6%; *F*(1,83) = 9.1; *p* < 0.005]. A main effect of experimental session was present in RTs [*F*(3,83) = 11.3; *p* < 0.0001], with responses being slower in the fMRI session (729 ms) than those in the three other experiments combined (620 ms).

More importantly, CSPC effects were clearly evident both in RTs [*F*(1,83) = 53.9; *p* < 0.0001] and error rates [*F*(1,83) = 11.4; *p* < 0.001] and were of comparable magnitude across experimental sessions [both *F*(3,83) < 1.8; n.s.]. Flanker interference effects were reduced for stimuli presented in the high-conflict location (RTs: 88 ms; error rates: 5.1%) relative to those in the low-conflict context (RTs: 121 ms; error rates: 7.2%; Figure 1B). Indicating that neither the lateralized response procedure introduced in data set III, nor informing participants about the contextual conflict-frequency manipulation in data set IV had any effect on CSPC effects, context × flanker congruency effects did not interact with the stimulus-response compatibility factor, experimental session, or their combination either in the RT or error rate data [all *F*(3,83) < 1.7; n.s.]. Replicating the finding that CSPC effects vary as a function of context transitions (King et al., 2012), they clearly occurred in context repetitions (47 ms), but were absent in context switches [19 ms; *F*(3,83) = 17.5; *p* < 0.0001]. Indicating the reliability of this effect, it did not vary across data sets [*F*(3,83) = 1.2; n.s.], even after excluding the data of our previous study [data set I; *F*(2,59) = 0.01; n.s.].

Together, the results of the conventional analyses of RTs and accuracy rates illustrate the robustness of CSPC effects on the one hand and an important boundary condition on the other, namely, that they appear to occur only in context repetitions. Additionally, they show that they are not confounded by spatial stimulus-response compatibility effects in the current paradigm and occur independently of participants’ awareness of the contextual conflict-frequency manipulation. However, these data do not speak to our motivating question whether CSPC effects reflect contextually cued adjustments in processing selectivity or shifts in response caution triggered by the infrequent events within each stimulus context. To address this issue, we turn now to the modeling data.

### Modeling data

The average parameter values for each of the four data sets for each of the four LBA models are shown in Table 1. Looking at the parameter values, it is apparent that, in general, the drift Models V1 and V2 accounted for CSPC effects by assuming that evidence accumulation rates were larger (i.e., faster) in the high-conflict context than in the low-conflict context on incongruent trials and smaller (i.e., slower) in the high-conflict context than in low-conflict contexts on congruent trials. The threshold Models B1 and B2, on the other hand, accounted for CSPC effects through the equivalent setting of response thresholds: larger thresholds in the high-conflict context than in the low-conflict context on congruent trials, and vice versa for incongruent trials.

**Table 1:** **Parameter values for Models V1 and V2 (“drift” models) and Models B1 and B2 (“threshold” models) averaged across participants in data sets I, II, III, and IV**.

	Data set	*s*	*A*	*b*_C-L_	*b*_I-L_	*b*_C-H_	*b*_I-H_	Δ*b*	*t*_0_	*v*_C-L_	*v*_I-L_	*v*_C-H_	*v*_I-H_	Δ*v*
Model V1	I	0.18	0.14		0.48			–	0.14	0.79	0.64	0.77	0.66	–
	II	0.15	0.09		0.36			–	0.13	0.78	0.63	0.75	0.65	–
	III	0.13	0.09		0.37			–	0.08	0.73	0.58	0.71	0.60	–
	IV	0.17	0.15		0.46			–	0.08	0.77	0.62	0.75	0.64	–
Model V2	I	0.18	0.14		0.48			–	0.14	0.79	0.64	–	–	0.02
	II	0.15	0.09		0.36			–	0.13	0.78	0.63	–	–	0.02
	III	0.13	0.10		0.37			–	0.08	0.73	0.59	–	–	0.02
	IV	0.17	0.15		0.46			–	0.08	0.77	0.62	–	–	0.02
Model B1	I	0.19	0.16	0.43	0.48	0.41	0.47	–	0.19	0.77	0.70	–	–	–
	II	0.17	0.10	0.32	0.36	0.33	0.35	–	0.17	0.76	0.70	–	–	–
	III	0.14	0.10	0.36	0.37	0.37	0.37	–	0.11	0.73	0.61	–	–	–
	IV	0.19	0.18	0.42	0.47	0.43	0.47	–	0.13	0.77	0.69	–	–	–
Model B2	I	0.19	0.17	0.43	0.48	–	–	0.01	0.20	0.78	0.70	–	–	–
	II	0.17	0.10	0.32	0.37	–	–	0.01	0.17	0.76	0.69	–	–	–
	III	0.14	0.11	0.36	0.37	–	–	0.01	0.12	0.75	0.62	–	–	–
	IV	0.18	0.20	0.43	0.47	–	–	0.01	0.14	0.79	0.70	–	–	–

*C, Con, I, Incon; H, High, L, Low; s, standard deviation; A, upper limit of the start-point distribution; b, response threshold; t_0_, non-decision time; v, drift rate. In Model B2, *b*_Incon-High_ = *b*_Incon-Low_ + Δ*b* and *b*_Con-High_ = *b*_Con-Low_ − Δ*b*. In Model V2, *v*_Incon-High_ = *v*_Incon-Low_ − Δ*v* and *v*_Con-High_ = *v*_Con-Low_ + Δ*v**.

### Observed and predicted RT distributions

**Figure 2:**
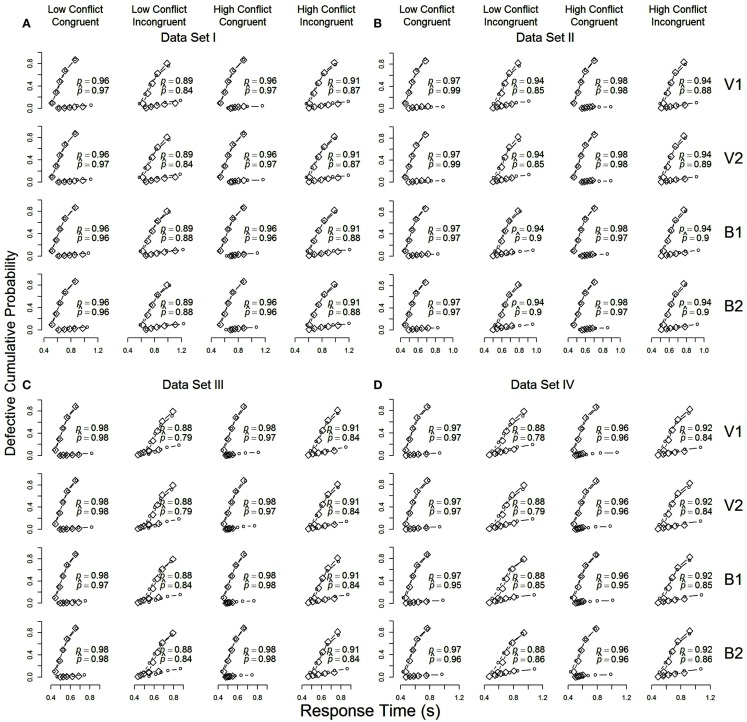
**Cumulative distribution function plots for data averaged over participants in each of the four data sets (A–D)**. Observed data (diamonds) and model predictions (circles) from Models V1 and V2 (“drift” models) and B1 and B2 (“threshold” models) are shown in the rows of each panel. For each condition (low- vs. high-conflict context, congruent vs. incongruent stimulus), the upper function presents results for correct response, and the lower function presents results for incorrect responses. For each condition, the observed and predicted proportion of correct responses are shown using *p* and $\hat{p}$ respectively.

The quality of agreement between the models and the data from each of the four data sets are plotted in Figures 2A–D (one figure per data set). The figure shows RT distributions for correct and erroneous responses on congruent and incongruent trials in the high- and low-conflict contexts (columns), along with model predictions from the four models (rows), as cumulative distribution function plots. Each plot is made up of quantile estimates from correct and incorrect RT distributions. The quantile estimates show the RT below which 10, 30, 50, 70, and 90% of the responses in that distribution fall. The positions of the quantiles on the *x*-axis reflect the speed at which responses are made, so that slower distributions stretch further to the right. The heights of the quantiles indicate, separately for correct and incorrect trials, the absolute cumulative proportion of responses with RTs below the quantile cutoff.

The plots in Figure 2 demonstrate that the predictions from all four models (circles) match the observed data (diamonds) well. The LBA model appears to give a good account of the full RT distributions for correct responses. All models appear to struggle somewhat to account for the speed of incorrect responses, especially in data sets III and IV (the lower function in each row of the second and fourth columns in Figures 2C,D). Differences between the models in their ability to fit the data are small, but perhaps most pronounced in their account of correct responses in low-conflict incongruent trials (the second column in each panel of Figure 2), particularly for data sets I and II. Models V1 and V2 (drift models) appear to systematically predict faster correct responses than were observed, while Models B1 and B2 (threshold models) also show this misfit, particularly for data sets III and IV, but to a lesser degree.

**Figure 3:**
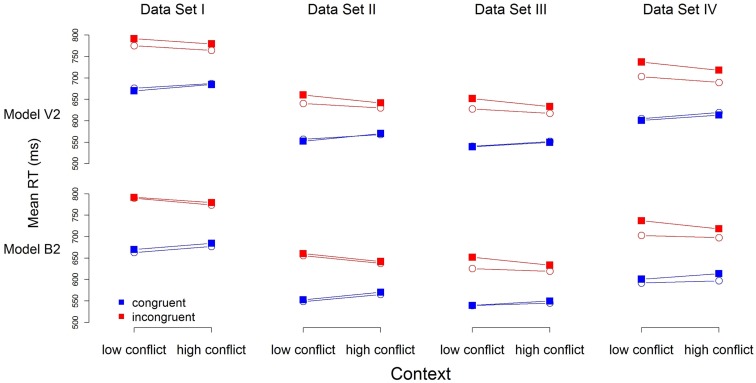
**Observed (filled squares) and predicted (open circles) mean RT for each of the four data sets**. Note: RTs were calculated in a manner similar to that in Figure 1B, with the exception that the first trial of each block was not excluded.

To help distinguish between the models, we turn to their predictions for mean RT. Figure 3 contains the predictions for mean RT for Models V2 and B2 (the predictions of Models V1 and B1 are very similar, and the overall pattern of misfits the same). Model predictions (open circles) are close to the observed data (filled squares) for both models. Model B2 does appear to outperform Model V2 for all but Data Set IV, for which both models appear to provide an equivalent account.

### Model selection

Table 2 contains AIC and BIC weights for each of the models for each of the four data sets. Additionally, the table presents the number of participants best fit by each model (in parentheses). The AIC weights suggest that Model B1 is most often the true model across participants and data sets (42.5% of the time), followed by Model B2 (38% of the time; threshold models), then Model V2 and finally V1 (the drift models “won” only roughly 20% of the time). The results are different using BIC, where we see that Model B2 is preferred more often than Model B1 (roughly 47 vs. 14% of the time). Notice also, however, that for data sets III and IV, the difference in model probabilities for Model B2 and V2 is less clear. The differences in conclusions drawn from BIC and AIC reflect the fact that BIC has a larger penalty for complexity, and that Model B2 has one fewer free parameter than Model B1, and because the response threshold models use one more free parameter than their respective drift models.

**Table 2:** **AIC and BIC weights for Models V1 and V2 (“drift” models) and B1 and B2 (“threshold” models) for each of the four data sets**.

		Data sets				Σ
		I	II	III	IV	
AIC	Model V1	0.059 (0)	0.050 (0)	0.185 (4)	0.152 (2)	6.9%
	Model V2	0.106 (4)	0.094 (2)	0.119 (3)	0.116 (2)	12.6%
	Model B1	0.412 (8)	0.498 (9)	0.468 (12)	0.419 (8)	42.5%
	Model B2	0.422 (13)	0.358 (8)	0.228 (6)	0.313 (6)	38.0%
	B vs. V	5.06	5.94	2.29	2.73	
BIC	Model V1	0.027 (0)	0.015 (0)	0.129 (3)	0.085 (1)	4.6%
	Model V2	0.313 (8)	0.221 (5)	0.389 (10)	0.337 (7)	34.5%
	Model B1	0.123 (3)	0.255 (4)	0.216 (4)	0.153 (1)	13.8%
	Model B2	0.537 (14)	0.509 (10)	0.267 (8)	0.425 (9)	47.1%
	B vs. V	1.94	3.24	0.934	1.37	

*The row labeled B vs. V shows how much more likely that either of Models B1 or B2 is the true model compared to Model V1 or V2 according to AIC and BIC. The values in parentheses represent the number of participants best fit by each model in each data set. The sum column (Σ) shows the percentage of participants for which each model provided best fit*.

The AIC and BIC weights can be used to compare the “B” model class, the response threshold models, to the models assuming that CSPC effects are due to changes in the evidence accumulation rate, the “V” model class (i.e., Models B1 and B2 vs. Models V1 and V2). The rows labeled “B vs. V” in Table 2 report how much more likely a response threshold model is the true model than a drift model. Averaged across data sets, a model assuming a response threshold-based explanation for CSPC effects is 1.87 times more likely to be the true model than the drift model according to BIC and four times more likely according to AIC. Thus, contrary to the hypothesis that CSPC reflect contextually cued adjustments in perceptual processing selectivity (e.g., Crump et al., 2006; Lehle and Hübner, 2008; Wendt et al., 2008; King et al., 2012), the current results obtained with the LBA model suggest that this phenomenon might be better attributed to shifts in response caution primed by infrequent events within each stimulus context.

## Discussion

We applied the LBA model to performance from four independent flanker task data sets to adjudicate between (1) the hypothesis that CSPC effects reflect adjustments in processing selectivity cued by contextual information associated with conflict frequency (Corballis and Gratton, 2003; Crump et al., 2006, 2008; Lehle and Hübner, 2008; Wendt et al., 2008; Crump and Milliken, 2009; Heinemann et al., 2009; Vietze and Wendt, 2009; Wendt and Kiesel, 2011; Bugg and Hutchison, 2012; D’Angelo and Milliken, 2012; King et al., 2012; Sarmiento et al., 2012; for review, see Bugg and Crump, 2012) and (2) an alternative account which attributes the phenomenon to shifts in response caution triggered by the occurrence of contextually unexpected events (e.g., incongruent trials in the low-conflict context). We predicted that if context-specific improvements in interference resolution index priming of attentional focus in favor of target stimuli, a model in which the *rate* of evidence accumulation (i.e., drift) was allowed to vary across context and congruency conditions would provide the best fit to the observed performance. In contrast, if contextual variation in the efficiency of conflict-control is attributable to shifts in response caution triggered by violations of expectancy about stimulus congruency (i.e., prediction errors), we assumed a model in which the *amount* of sensory evidence required to reach a decision (i.e., response threshold) varied according to event frequency within each context would deliver the best explanation of the empirical data. We implemented two “drift” and “threshold” models to account for CSPC effects. The results showed that while both classes of models captured the observed performance well (at least for correct trials), models explaining CSPC effects as resulting from shifts in response caution (i.e., the threshold models) accounted for the data better than those attributing the phenomenon to adjustments in processing selectivity (i.e., the drift models). Although evidence indicating that the threshold models provided better fit than the drift models ranged from only relatively weak (as expressed in BIC) to moderately strong (as expressed in AIC), the differences in the model selection parameters between the two classes of models were fairly consistent across the four data sets. Together, these findings provide preliminary evidence that the currently dominant view of the mechanisms underlying CSPC effects may need to be reconsidered.

Previous behavioral investigations of CSPC effects have suggested that the presentation of a stimulus in a context associated with frequent conflict primes the retrieval and execution of contextually appropriate conflict-control settings, facilitating interference resolution by enhancing processing selectivity (Corballis and Gratton, 2003; Crump et al., 2006, 2008; Lehle and Hübner, 2008; Wendt et al., 2008; Crump and Milliken, 2009; Heinemann et al., 2009; Vietze and Wendt, 2009; Wendt and Kiesel, 2011; D’Angelo and Milliken, 2012; Sarmiento et al., 2012). Our recent fMRI study corroborated this “priming of control” hypothesis by showing that CSPC effects were mediated by activity in a region of the mSPL demonstrated to be involved in attentional control (e.g., Yantis, 2008; Shomstein, 2012) and that this activity explained top-down modulation of task-related sensory processing in visual cortex (King et al., 2012). The current modeling results qualify these previous interpretations, however, because they suggest that CSPC effects may reflect more a consequence of a shift in decision criterion triggered by contextually unexpected events than adjustments in attentional focus driven by conflict frequency.

Further insight into the putative origin of CSPC effects can be gained by considering the current results vis-à-vis those obtained by analyzing CSPC effects as a function of context transitions (i.e., context switches vs. repetitions) in our previous fMRI study. Specifically, we found in that study that while CSPC effects were observable in mSPL activation immediately upon a switch between contexts, they were observable in behavior only after context repetitions (a finding replicated here across all four data sets), suggesting that contextually appropriate control settings are rapidly retrieved in a highly flexible manner and mediate behavioral adaptation on the following trial(s) in that context (King et al., 2012). Although an analogous analysis with the LBA could not be conducted here due to an inadequate number of trials after splitting up the context and congruency conditions according to the context transition factor, it can be assumed that the mechanism suggested to mediate CSPC effects by the current modeling results (i.e., prediction error-triggered adjustments in response threshold) is also driven by context repetitions and not by context changes, in particular because CSPC effects were present only when context repeated in all experiments. It thus seems reasonable to speculate that adjustments in response threshold triggered by unexpected events would require at least one context repetition in order for a prediction regarding upcoming stimulus congruency to be in place. This view implies that contextual conflict-control settings entail predictions regarding upcoming congruency such that the relative performance gain for contextually likely stimuli (e.g., incongruent trials in the high-conflict context) and the relative performance decrement for contextually unlikely stimuli (e.g., incongruent trials in the low-conflict context) which comprise CSPC effects reflect fulfillment and violation of expectations, respectively. Such a proposition would be generally in line with the notion that the cognitive system promotes processing efficiency and goal-directed performance by continuously generating models of the environment according to current contextual demands and information stored in memory to predict future stimulus input (Friston, 2005). In any event, this novel perspective on the putative origin of CSPC effects would not have been possible from traditional analyses of behavior or functional neuroimaging alone.

It should be noted, however, that the present results provide only tentative evidence for notion that CSPC effects reflect prediction error-triggered adjustments in response caution, and some caveats should be kept in mind when interpreting our data. First, even though the LBA provided reliably good fit to the current empirical data, the model was not originally conceived to account for behavior on tasks in which the information being accumulated changes in quality over time. Many of the current theories for the flanker task assume, however, that an attentional window narrows in on the target stimulus either gradually (Eriksen and St James, 1986; Cohen et al., 1992) or abruptly (Hübner et al., 2010) over the course of a trial, thus improving the quality of evidence as time progresses. This is in direct contrast to the fundamental assumption of the LBA that evidence accumulation rate is constant over time (Brown and Heathcote, 2008). Second, the current modeling effort is at odds with another basic assumption of sequential sampling models, namely, that response threshold is already set prior to evidence accumulation. By contrast, both of the favored Models B1 and B2 captured CSPC effects by letting response threshold be adjusted according to stimulus congruency. This leads to the theoretically problematic proposition that congruency is already “known” before the start of evidence accumulation. Nevertheless, it could be argued that these models are in principle feasible, in particular because the stimuli in the present studies do not need to be analyzed to a high degree in order to distinguish congruent from incongruent trials, given the pronounced perceptual difference between congruent and incongruent arrays at a Gestalt level (cf. Baylis and Driver, 1992). Moreover, the detection of stimulus congruency (or of a stimulus as being perceptually surprising) as such is of no help in deciding whether the target face is oriented to the left or right. Therefore, the assumption that congruency or stimulus Gestalt can be detected (and affect threshold settings) *before* the decision-making process regarding target face orientation has been completed is not implausible. In sum, shifts in response threshold could feasibly be driven by a fast perceptual oddball detection occurring immediately following initial encoding of lower-level stimulus attributes, but prior to any in-depth stimulus processing or categorization according to a higher-level criterion such as target face orientation.

In future research, we aim to explore whether results similar to those reported here would be delivered by recent adaptations of sequential sampling models that were designed specifically to accommodate decision making in flanker tasks and avoid the issues outlined above, such as the spotlight diffusion model (White et al., 2011). We did not use White et al.’s model in the current analysis simply because it was not practically possible for us to achieve optimal model fits to the near 100 individual participants for all model parameterizations within a reasonable time period. The advantage of a time-varying rate of evidence accumulation in White et al.’s model is clear, but since it must be simulated (involving under optimal computing conditions several hours per model per subject), we opted to use the more computationally efficient LBA model (requiring less than a minute per model per subject) for the current project. One might speculate that a diffusion-like model in which drift rate can rapidly accelerate or decelerate within-trials as a function of fulfillment or violation of contextual expectancies regarding stimulus congruency would provide a better account of CSPC effects than the favored threshold models as revealed here with the LBA.

It is promising nonetheless that despite the LBA’s possible mis-specification, the model provided good fit to the observed RTs across data sets, at least on correct trials. Although we cannot rule out that the relative misfit for error trial RT distributions was not a consequence of the violations outlined above, we speculate that it may be attributable to the overall high performance/relatively low error rates and fast error RTs in the current experiments. That is, since the fast error RTs did not occur in all subjects or data sets, it is unclear to what extent they are reliable and should be used to discount the applicability of a model like the LBA. Future studies using similar protocols might create conditions that are more error prone, for instance, by reducing the stimulus presentation time. Such data would help determine whether the misfits here are simply an artifact of the fitting method, or reveal a true misfit of the model.

If we take the present results at face value, however, they provide initial support for an intriguing alternative account of CSPC effects. According to this new hypothesis, subjects implicitly encode the stimulus statistics (i.e., frequency of different trial types) associated with each context, just like in the currently dominant view of the phenomenon. However, instead of selectively enhancing their attentional focus to stimuli presented in the high-conflict context, they may form perceptual expectations for the frequent trial types in both the high- and low-conflict contexts (presumably to optimize perceptual inference and/or response selection). When expectations in a given context are violated, perceptual prediction errors in visual cortex may then be used as a control signal, indicating the need to raise response thresholds, such that sufficient evidence can be accumulated about the unexpected stimulus and a correct response can be selected. The notion that visual processing underlying perceptual inference is strongly driven by expectations and prediction error signals has garnered much empirical support in recent years (Summerfield and Koechlin, 2008; Summerfield et al., 2008; Egner et al., 2010; Jiang et al., 2012), thus supporting the basic neural feasibility of this hypothesis. Convergent electroencephalographic and fMRI evidence suggests that a subcortical-frontomedial network including the anterior mid-cingulate cortex, a region traditionally thought to be centrally involved conflict- and error monitoring (Botvinick et al., 2001; Ridderinkhof et al., 2004), may drive the type of adaptation investigated here by responding, more generally than to conflicts or errors *per se*, to any unexpected event and evaluating whether adjustments are needed (Wessel et al., 2012), confirming the core predictions of recent computational modeling work (Alexander and Brown, 2011; Silvetti et al., 2011; see also Egner, 2011). Note that, under this new perspective, CSPC effects can still be argued to constitute a reflection of “priming of control” (Spapé and Hommel, 2008; Verguts and Notebaert, 2008, 2009; King et al., 2012), but the nature of the primes and control processes differ from previous assumptions, in that they represent a shift in response caution primed by contextually surprising stimuli rather than shifts in attentional focus primed by contextual cues.

In a related literature on item-specific proportion congruent (ISPC) effects (Jacoby et al., 2003; Blais et al., 2007), there has been some debate about whether improved interference resolution for mostly incongruent items reflects a selective conflict-control mechanism that enhances processing for specific items, or merely an associative, contingency-based process by which participants learn associations between salient distracter features and responses (Schmidt and Besner, 2008; Bugg et al., 2011; Bugg and Hutchison, 2012). Crump and Milliken (2009) and Heinemann et al. (2009) both demonstrated that CSPC effects are immune to a purely associative account, because they generalize to frequency-unbiased “transfer” items. The CSPC effects in the current experiments underline these previous findings and provide further support for a control account, because they were obtained using trial-unique stimuli (i.e., the identity of the faces in the flanker array was novel on each trial) and neither stimulus congruency nor conflict-frequency context were predictive of a specific response.

Validation of the current results and their potential impact on theories of conflict-control will involve various lines of future research. First, although our modeling results were more or less consistent across all four data sets, it remains to be seen whether a “threshold” model would also provide a better account for CSPC effects than a “drift” model in other interference paradigms, such as the Stroop task. Second, as detailed above, both the experimental tasks and computational modeling approaches have scope for additional optimization for further addressing the question asked here. Additionally, new empirical protocols could be developed to provide a direct test of the notion that CSPC effects reflect increased response caution elicited by prediction errors.

In conclusion, this study suggests that CSPC effects may not necessarily reflect contextually cued attentional focus as commonly conceived, but rather shifts in response caution triggered by contextually surprising stimuli. While generally in line with the “priming of control” hypothesis (Spapé and Hommel, 2008; Verguts and Notebaert, 2008, 2009; King et al., 2012), it should be reiterated that this is the first attempt to use a model of choice and RT distributions to account for CSPC effects and more research with specialized modeling techniques that avoid the potential drawbacks of our LBA-based approach is needed to corroborate this novel perspective. If valid, the notion that expectancy violations can drive conflict adaptation effects, regardless of whether they are context-specific as in the current study, or on an item-specific (e.g., Blais et al., 2007) or sequential level (e.g., Egner, 2007), would bring important insight on the mechanisms underlying conflict-control.

## Conflict of Interest Statement

The authors declare that the research was conducted in the absence of any commercial or financial relationships that could be construed as a potential conflict of interest.
